# Factors associated with arterial stiffness assessed by pulse pressure amplification in healthy children and adolescents: a cross-sectional study

**DOI:** 10.1186/s12887-023-03942-1

**Published:** 2023-04-03

**Authors:** Leticia Pereira Salomão, Giselle Santos Magalhães, José Felippe Pinho da Silva, Luzia Maria dos Santos, Isabel Cristina Gomes Moura, Bruno Almeida Rezende, Maria Glória Rodrigues-Machado

**Affiliations:** Programa de Pós-Graduação em Ciências da Saúde - Faculdade Ciências Médicas-MG, Alameda: Ezequiel Dias, n 275. Bairro: Centro, CEP 30130-110 Belo Horizonte/MG, Brasil

**Keywords:** Pulse pressure, Aortic pulse wave, Central systolic blood pressure, Arterial stiffness

## Abstract

**Background:**

Increasing evidence suggests that reducing pulse pressure amplification (PPA) plays an important role in pathogenesis and progression of cardiovascular disease. This is a cross-sectional, observational, and analytical study in which we evaluated the associated factors with a greater chance of reducing PPA in 136 healthy children and adolescents aged 8 to 19 years old stratified by gender and age group.

**Methods:**

Arterial stiffness and vascular and hemodynamic parameters were non-invasively measured using Mobil-O-Graph® (IEM, Stolberg, Germany), a cuff-based oscillometric device. PPA was expressed as the peripheral-to-central pulse pressure ratio (PPp / PPc). Participants with PPA < 1.49 were considered as part of the arterial stiffness group.

**Results:**

In a univariate model, the increase in total vascular resistance, the reflection coefficient and the augmentation pressure were more likely to have arterial stiffness in all groups. The factors most likely to have arterial stiffness (as assessed by the reduction of the PPA) in the multivariate model were increasing age, the reflection coefficient and cardiac index in the total sample, male group and child and adolescent groups. In addition to age in the female group, cardiac output, stroke volume, and AIx@75 were the factors most likely to present arterial stiffness.

**Conclusions:**

The results show for the first time in children and adolescents that the factors most likely to reduce PPA are related to the reflection wave, which determines aortic pressures and, therefore, left ventricular afterload.

## Background

Arterial stiffness is a natural aging process that can be accelerated by the cumulative effects of risk factors such as high blood pressure, hyperglycemia, adiposity, hypercholesterolemia [1], sedentary lifestyle [2] and chronic inflammation [3]. Many of these risk factors are increased in children and adolescents due to a sedentary lifestyle and atherogenic eating habits. The consumption of foods rich in simple sugars and fat leads to obesity, hypertension, type 2 diabetes or metabolic syndrome. Therefore, sensitive, and non-invasive tools are needed to detect early preclinical changes in the arteries.

Arterial stiffness can be assessed by invasive and non-invasive methods. However, there is a lack of validation studies in pediatrics comparing invasive measures such as cardiac catheterization and non-invasive measures due to the ethical concerns involved with invasive studies in children for research purposes only [4]. The most commonly used methods are pulse wave velocity (PWV), augmentation index (AIx, AIx@75 corrected by the heart rate of 75 bpm), central pulse pressure (PPc) and systolic blood pressure SBPc [5]. PWV is assessed between the carotid and femoral arteries and is considered the gold standard for measuring arterial stiffness. AIx@75 provides information on the role played by reflection waves in determining PPc. In addition to PWV, PPc, SBPc and AIx@75, pulse pressure amplification (PPA) is also considered an arterial stiffness index. PPA in healthy adults gradually increases from the aorta/carotid arteries to the brachial/radial arteries. This phenomenon is mainly attributed to the gradual increase in SBPc from the aorta to the periphery due to the reflection wave [6, 7]. With aging, or in diseases that progress with arterial stiffness, the central arteries become stiff earlier than the brachial arteries, leading to a change in the amplitude and contour of the central pressure wave. Consequently, PPc increases more than peripheral PP (PPp), and physiological PPA is attenuated because of the reduction in the difference between the two PP [8, 9].

Studies in adults show that PPA has a significant inverse relationship between age and its reduction has a negative impact on mortality, cardiovascular events, and damage to noble organs such as the heart, kidney and brain [1, 10]. The number of PPA studies in children and adolescents is small and with a limited number of participants. Mukarami et al. were the first to study PPA in children and adolescents. These authors observed that PPA is present in children, but contrary to what happens in adulthood, it increases during childhood [11]. These same authors also showed a reduction in PPA in patients after aortic arch repair when compared to the control group [12] and after surgery for complete transposition of great arteries [13]. A limitation of these studies is that they were performed on anesthetized patients. A recent study from our group showed that children and adolescents with type I diabetes had lower PPA when compared to children considered healthy, matched for gender and age [14]. In this study, associations between PPA and various indices of arterial stiffness in children and adolescents with type I diabetes were observed. PPA negatively correlated with total vascular resistance (TVR), augmentation pressure and reflection coefficient, and positively with cardiac index. These results support the concept that there is accelerated arterial aging in this population, and may explain, at least in part, the increased cardiovascular risk in these patients [14]. Lydakis et al. (2012) demonstrated a negative association between body mass index (BMI) and PPA in overweight and obese children and adolescents [15].

Although the atherosclerotic process often starts early in childhood, the clinical manifestations of cardiovascular disease (CVD) appear in adulthood [16]. Thus, there is a great need to assess vascular dysfunction markers in the pediatric population. In turn, the aim of the present study was to evaluate and identify predictors of reduced PPA in children and adolescents considered healthy, categorized by gender and age. Our hypothesis was that the attenuation of PPA may be associated with other better-established arterial stiffness indices, as well as the habitual physical activity level, quality of life, socioeconomic level and anthropometric data in children and adolescents.

## Methods

This is a cross-sectional study to assess PPA in children from 8 years old and adolescents up to 19 years old, considered healthy, with normal body mass index (BMI). Children and adolescents with acute or chronic diseases were excluded from the study. A total of 177 students were recruited from public schools in 9 metropolitan regions of Belo Horizonte. However, 41 participants were excluded for the following reasons: 8 did not show up on the day scheduled for the exam, 9 did not sign the Informed Parental Consent Form or the Assent Form, 17 had a history of chronic respiratory disease (recurrent wheezing or diagnosis of asthma), 3 used medications for epilepsy, 2 were using Ritalin and 2 had a high BMI. Thus, the final sample consisted of 65 female and 71 male participants.

### Sample size calculation

The sample size was calculated according to Duarte et al. [14]. The sample of 136 children and adolescents presented a significance level of 5% and power of more than 80%. The calculation was performed using the Open-epi version 3.01 program.

### Experimental protocol

After parents or guardians provided written informed consent, the participants answered the questionnaires to assess their socioeconomic level, quality of life and habitual physical activity level. The questionnaires were self-administered and when participants had difficulty understanding an item, they were helped by the trained researchers. Anthropometric data were subsequently collected, followed by cardiovascular measurements.

### Anthropometric assessment

BMI was obtained by the ratio between weight (kg) and height squared (m^2^). Children and adolescents with percentiles between 5 and 85% were included in the study. Waist (WC) and hip (HC) circumferences were measured and the ratios between WC/Height and WC/HC were obtained. Cut-off points based on WHO guidelines (2007) were used to analyze weight, height, and BMI [16]. WC/HC is one of the criteria used to characterize the metabolic syndrome and cardiovascular risk, with cut-off values of 0.90 for men and 0.85 for women [17].

### Assessment of habitual physical activity level

The short version of the Physical Activity Questionnaire-Child (IPAQ-C) was used to assess habitual physical activity level, which investigates the practice of physical activity in children and adolescents in the seven days prior to filling it out. It consists of nine questions related to physical activity performed at school and during leisure time during the week and on weekends. It also includes questions related to the time spent in front of the television and the computer. Each question has scores from 1 to 5. Those who spent 150 min performing moderate physical activities during the week were considered active [18].

### Quality of life assessment

Health-related quality of life was evaluated using the Pediatric Quality of Life Inventory Version 4.0 (Peds-QL 4.0) [19]. It is a questionnaire consisting of 23 questions that assess the perception of children and adolescents, and is divided into two domains: physical (8 items), emotional—cognitive and intellectual (5), social (5 items), and school (5 items). Items were rated on a scale of points from zero to 100; where zero = 100 points; one = 75; two = 50; three = 25; four = 0. Then, the average of these items was measured. The higher the score, the better the perception of quality of life.

### Respiratory health condition assessment

The assessment of respiratory health status was assessed using the International Study of Asthma and Allergies in Childhood (ISAAC) questionnaire, with the aim of excluding volunteers with a significant respiratory history or medical diagnosis of asthma, as this is a very prevalent disease in this age group [20]. This questionnaire contains 12 questions that assess the presence of hissing and wheezing in the past 12 months or over a lifetime. The higher the score attributed to the questions, the greater the chances of the individual having asthma or other respiratory system impairments.

### Socioeconomic classification criteria

The socioeconomic classification was performed as recommended by the Brazilian Association of Research Companies [21]. The socioeconomic questionnaire used in this study presents questions related to the quantity of assets, education, and income of the head of the family. A score was assigned to each selected item and the final result was used to classify participants either into social class A, A + B, C, C + D, or E. This classification was used in this study only to characterize the sample.

### Arterial stiffness assessment

**Fig. 1:**
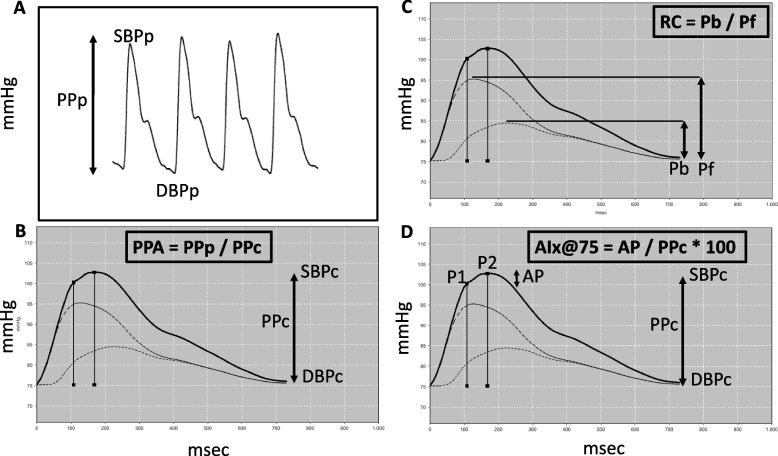
Pulse waves from the brachial (**A**) and aortic (**B **- **D**) arteries in a male teenager. **A **Peripheral pulse wave (measured). SBPp and DBPp = Peripheral systolic and diastolic blood pressure; PPp = Peripheral pulse pressure. **B** Central pulse wave (calculated). SBPc and DBPc = Central systolic and diastolic blood pressure; PPc = Central pulse pressure; PPA = Pulse pressure amplification (PPp / PPc). **C** Pf = Forward or ejection wave; Pb = Backward or reflection wave; RC – Reflection coefficient. **D **P1 = First systolic peak; P2 = Second systolic peak, AP = Augmentation pressure (P2 – P1), AIx@75 = Augmentation index corrected for a heart rate of 75 bpm

Cardiovascular parameters were performed according to previous study by our group [14]. The Mobil-O-Graph® Pulse Wave Analysis Monitor (Mobil-O-Graph, IEM, Germany) was used to assess arterial stiffness indices, vascular and hemodynamic parameters. This device uses brachial oscillometric blood pressure (Fig. 1A) for a non-invasive estimate of SBPc and aortic pulse wave velocity (PWV) measurement. The aortic pulse wave (Figs. 1B,1C, 1D) is calculated from the brachial arterial pressure, and is the sum of the incident wave generated by the contraction of the left ventricle (forward wave, Pf) and the reflection wave (backward wave, Pb) coming from the periphery (Fig. 1C). The increase in SBPc due to the reflection wave (augmentation pressure—AP), expressed as a percentage of PPc, corresponds to AIx@75, i.e. AIx@75 = AP / PPc * 100 (Fig. 1D). PWV is calculated by a mathematical model considering various parameters of the aortic pulse wave and wave separation analysis. PPA was expressed as the peripheral-to-central PP ratio (Fig 1B) [22]. The device performs three consecutive measurements and the mean of the three was considered for the final analysis. In addition to arterial stiffness indices, peripheral and central blood pressure, hemodynamic parameters are also provided (systolic volume, cardiac output, cardiac index and total vascular resistance).

The cuffs were selected according to the circumference of the participant’s left arm, where the measurements were taken. The measurement was performed with the participant sitting, in a calm and quiet place, after a minimum rest of 5 min. The room temperature was maintained between 21 degrees according to the manufacturer's recommendation. The researchers responsible for data collection were trained to handle the Mobil-O-Graphy and apply the questionnaires.

All participants were instructed to maintain a postprandial state of at least three hours and 24 h of abstaining from physical exercise, consumption of caffeine or other stimulant food before this stage.

The use of Mobil-O-Graphy to assess cardiovascular parameters in children and adolescents has previously been used by our research group [14, 23, 24] and other groups [25, 26]. Shiraishia et al. (2019) recently validated the use of Mobil-O-Graphy to assess SBPc in children and adolescents [27].

### Statistical analysis

The variable PPA was categorized into values less than and greater than or equal to the median (1.49). The group of children and adolescents with PPA < 1.49 was called the arterial stiffness group, and with PPA ≥ 1.49 as the control group.

Categorical variables were presented as counts and percentages, and numerical variables as mean ± standard deviation (median). Numerical variables were submitted to the Shapiro–Wilk normality test.

Univariate logistic regression models were built to assess the association between each of the analyzed variables and arterial stiffness indices. The results were presented as odds ratio (OR) and the respective p-values for the whole sample, and stratified by gender and age group. Variables with *p* < 0.20 in the univariate analysis were included in a saturated model, to which the final model was reached using the backward strategy, with variables with *p* < 0.05 and the variable age independent of significance to control effects. The results were presented as OR, 95% confidence interval of the OR and respective p-values. The quality of the fit was analyzed using the Hosmer–Lemeshow (HL) test. The analysis was developed in the free R version 4.0.2 program.

## Results

The study involved 136 individuals, 81 (59.6%) adolescents and 71 (52.2%) males. Moreover, 62 participants (45.6%) were in social class A or B, and 74 (54.4%) in social class C. Regarding the habitual physical activity level, 95 participants (69.8%) were considered active and 41 participants (30.2%) were considered sedentary. Table 1 presents the descriptive statistics of the anthropometric data, quality of life and cardiovascular variables of peripheral and central arterial pressure, hemodynamics and arterial stiffness indices of the sample.

**Table 1:** Anthropometric characteristics, physical activity profile, quality of life, parameters of peripheral and central arterial pressure, hemodynamic parameters and arterial stiffness of the individuals in the sample

Variables	Sample (*n* = 136)
Gender M	71 (52.2%)
Age (years)^a^	13.45 ± 3.21 (14)
Weight (kg)^a^	46.94 ± 12.60 (47)
Height (cm)^a^	155.63 ± 14.95 (156.50)
BMI (kg/m^2^)^a^	18.92 ± 2.43 (18.60)
WC (cm)^a^	71.89 ± 10.57 (70.75)
HC (cm)^a^	78.74 ± 12.90 (78)
WC/HC^a^	0.87 ± 0.18 (0.81)
WC/Stature^a^	0.45 ± 0.07 (0.44)
**Quality of Life**	
Physical score^a^	84.32 ± 13.33 (85.94)
Emotional score^a^	71.45 ± 20.72 (75)
Social score^a^	88.07 ± 12.18 (90)
Educational score^a^	79.08 ± 15.89 (80)
**Peripheral arterial pressure**	
SBPp (mmHg)	111.48 ± 8.16 (111.17)
DBPp (mmHg)	69.50 ± 6.16 (69.67)
MAPp (mmHg)	88.13 ± 8.05 (87.67)
PPp (mmHg)	42.21 ± 7.37 (41.67)
**Central blood pressure**	
SBPc (mmHg)	98.75 ± 6.86 (97.83)
DBPc (mmHg)	70.94 ± 6.19 (71.17)
PPc (mmHg)	28.09 ± 5.03 (28)
Pulse pressure amplification (PPA)	1.53 ± 0.17 (1.49)
**Hemodynamics**	
Systolic volume (ml)	56.94 ± 11.37 (56.09)
Cardiac output (l/min)	4.45 ± 0.51 (4.38)
Total vascular resistance (s*mmHg/ml)	1.22 ± 0.11 (1.24)
Cardiac index (l/min*1/m^2^)	3.23 ± 0.59 (3.17)
Heart rate (bpm)	79.48 ± 11.45 (80.17)
**Arterial stiffness**	
Augmentation pressure (mmHg)	5.88 ± 2.63 (5.67)
AIx@75 (%)	22.30 ± 7.94 (22.17)
PWV (m/s)	4.54 ± 0.27 (4.50)

^a^Data expressed as mean ± standard deviation (median). *BMI* Body mass index,  *WC* Waist circumference,  *HC Hip circumference,* *SBP* Systolic blood pressure, *DBP* Diastolic blood pressure, *MAP* Mean arterial pressure, *PP* Pulse pressure, *AIx@75* Augmentation index corrected for heart rate of 75 bpm PWV Pulse wave velocity

The minimum and maximum PPA values were 0.95 and 2.05, respectively. The coefficient of variation was 12.45%. Anthropometric data, socioeconomic class, habitual physical activity level, and quality of life were not associated with a greater chance of having arterial stiffness, as assessed by PPA.

Table 2 shows the univariate logistic regression models to assess factors associated (anthropometric data, socioeconomic class, habitual physical activity level and quality of life) with lower PPA values.

**Table 2:** Univariate logistic regression models to assess factors associated with lower pulse pressure amplification values

Variables	Sample (*n* = 136)	Gender		Age Group	
		Male (*n* = 71)	Female (*n* = 65)	Child (*n* = 55)	Adolescent (*n* = 81)
Gender					
Male	-	-	-	-	-
Female	0.94 (*p* = 0.86)	-	-	0.54 (*p* = 0.27)	1.41 (*p* = 0.44)
Age (years)	0.92 (*p* = 0.14)	0.87 (*p* = 0.07)	0.99 (*p* = 0.91)	1.18 (*p* = 0.54)	1.03 (*p* = 0.82)
Weight (kg)	1.00 (*p* = 0.91)	1.03 (*p* = 0.19)	0.97 (*p* = 0.20)	1.01 (*p* = 0.55)	1.00 (*p* = 0.94)
Height (cm)	1.00 (*p* = 0.86)	1.00 (*p* = 0.76)	0.99 (*p* = 0.55)	1.01 (*p* = 0.65)	1.00 (*p* = 0.80)
BMI (kg/m^2^)	1.02 (*p* = 0.76)	1.19 (*p* = 0.08)	0.85 (*p* = 0.13)	1.08 (*p* = 0.58)	1.02 (*p* = 0.84)
WC (cm)	1.01 (*p* = 0.32)	1.05 (*p* = 0.07)	0.99 (*p* = 0.67)	1.03 (*p* = 0.17)	1.00 (*p* = 0.85)
HC (cm)	1.01 (*p* = 0.70)	1.02 (*p* = 0.30)	0.99 (*p* = 0.61)	1.02 (*p* = 0.50)	1.01 (*p* = 0.60)
WC/HC	1.20 (*p* = 0.85)	0.56 (*p* = 0.63)	4.35 (*p* = 0.37)	3.17 (*p* = 0.43)	0.38 (*p* = 0.48)
WC/Stature	11.69 (*p* = 0.32)	62.39 (*p* = 0.27)	2.57 (*p* = 0.78)	49.90 (*p* = 0.26)	1.02 (*p* = 0.99)
Socioeconomic class					
A or B	-	-	-	-	-
C	1.13 (p = 0.73)	0.67 (*p* = 0.40)	2.00 (*p* = 0.17)	1.20 (*p* = 0.74)	1.12 (*p* = 0.80)
Physical Activity Profile					
Active	-	-	-	-	-
Sedentary	1.42 (*p* = 0.35)	2.58 (*p* = 0.06)	0.62 (*p* = 0.42)	1.22 (*p* = 0.73)	1.46 (*p* = 0.45)
Quality of life					
Physical Aspect	1.00 (*p* = 0.74)	1.00 (*p* = 0.92)	0.99 (*p* = 0.70)	0.97 (*p* = 0.13)	1.02 (*p* = 0.27)
Emotional Aspect	0.99 (*p* = 0.32)	0.99 (*p* = 0.43)	0.99 (*p* = 0.56)	0.98 (*p* = 0.14)	1.00 (*p* = 0.90)
Social Aspect	1.00 (*p* = 0.73)	1.00 (*p* = 0.99)	1.01 (*p* = 0.65)	0.99 (*p* = 0.81)	1.02 (*p* = 0.40)
Educational Aspect	1.00 (*p* = 0.94)	1.01 (*p* = 0.55)	0.99 (*p* = 0.56)	0.98 (*p* = 0.18)	1.01 (*p* = 0.36)

Data expressed as OR and *p*-value. *BMI* body mass index, *WC* waist circumference, *HC* hip circumference

### Whole sample

An increase in TVR (*p* < 0.01), AIx@75 (*p* = 0.01), reflection coefficient (*p* < 0.01) and augmentation pressure in the univariate analysis for the entire sample represented a greater chance of having arterial stiffness (*p* < 0.01). On the other hand, an increase in cardiac output (*p* < 0.01) and cardiac index (*p* = 0.03) presented less chance of increasing arterial stiffness (Table 3). In addition, increasing age (*p* = 0.016) and cardiac index (*p* < 0.001) in the multivariate model were associated with a lower chance of having arterial stiffness, and an increase in reflection coefficient was associated with a higher chance of increasing PPA (*p* < 0.001) (Table 4).

**Table 3:** Univariate logistic regression models to assess factors associated with lower pulse pressure amplification values

Variables	Sample (*n* = 136)	Gender		Age Group	
		Male (*n* = 71)	Female (*n* = 65)	Child (*n* = 55)	Adolescent (*n* = 81)
**Hemodynamics**					
SV (ml)	0.99 (*p* = 0.43)	1.00 (*p* = 0.82)	0.97 (*p* = 0.18)	1.01 (*p* = 0.70)	0.97 (*p* = 0.20)
CO (l/min)	0.30 (*p* < 0.01)	0.48 (*p* = 0.15)	0.16 (*p* < 0.01)	0.46 (*p* = 0.12)	0.18 (*p* < 0.01)
TVR (s*mmHg/ml)	415.23 (*p* < 0.01)	125.03 (*p* = 0.03)	5.21e + 03 (*p* < 0.01)	191.92 (*p* = 0.06)	675.24 (*p* < 0.01)
CI (l/min*1/m^2^)	0.52 (*p* = 0.03)	0.48 (*p* = 0.06)	0.58 (*p* = 0.29)	0.55 (*p* = 0.18)	0.45 (*p* = 0.07)
**Arterial stiffness**					
RC	1.35 (*p* < 0.01)	1.24 (*p* < 0.01)	1.60 (*p* < 0.01)	1.41 (*p* < 0.01)	1.36 (*p* < 0.01)
AP (mmHg)	1.49 (*p* < 0.01)	1.35 (*p* = 0.01)	1.70 (*p* < 0.01)	1.67 (*p* < 0.01)	1.46 (*p* < 0.01)
PWV (m/s)	0.65 (*p* = 0.51)	1.72 (*p* = 0.58)	0.28 (*p* = 0.17)	0.79 (*p* = 0.83)	0.74 (*p* = 0.71)
AIx@75 (%)	1.06 (*p* = 0.01)	1.01 (*p* = 0.77)	1.13 (*p* < 0.01)	1.03 (*p* = 0.44)	1.09 (*p* < 0.01)

Data expressed as OR and *p*-value. *SV* Systolic volume, *CO* Cardiac output, *TVR* Total vascular resistance, *CI* Cardiac index, *RC* Reflection coefficient, *AP* Augmentation pressure, *PWV* Pulse wave velocity, *AIx@75* Augmentation index corrected for heart rate of 75 bpm

**Table 4:** Multivariate logistic regression models to assess factors associated with arterial stiffness assessed as lower pulse pressure amplification values

Variables	OR	CI 95%	*P*-value
**The whole sample (HL *p*-value 0.369)**			
Constant	8.86e-07	(4.36e-10; 4.97e-04)	< 0.001
Age (years)	0.81	(0.68; 0.96)	0.016
Cardiac index (l/min*1/m^2^)	0.14	(0.05; 0.36)	< 0.001
Reflection coefficient	1.48	(1.31; 1.74)	< 0.001
**Male (HL *p*-value 0.839)**			
Constant	2.34e-05	(1.53e-09; 0.08)	0.016
Age (years)	0.78	(0.61; 0.96)	0.026
Cardiac index (l/min*1/m^2^)	0.10	(0.02; 0.33)	< 0.001
Reflection coefficient	1.44	(1.23; 1.79)	< 0.001
**Female (HL *p*-value 0.193)**			
Constant	0.02	(8.9e-07; 188.59)	0.384
Age (years)	0.90	(0.70; 1.14)	0.413
Cardiac output (l/min)	0.03	(2.48e-03; 0.24)	0.003
Systolic volume (ml)	1.37	(1.17; 1.69)	< 0.001
AIx@75 (%)	1.28	(1.12; 1.52)	0.001
**Child (up to 12 years old) (HL *p*-value 0.468)**			
Constant	9.86e-09	(3.20e-16; 1.71e-03)	0.011
Age (years)	1.22	(0.59; 2.68)	0.592
Cardiac index (l/min*1/m^2^)	0.20	(0.04; 0.80)	0.036
Reflection coefficient	1.48	(1.24; 1.94)	< 0.001
**Adolescents (13 to 19 years old)** **(HL *p*-value 0.441)**			
Constant	2.40e-09	(7.98e-16; 2.28e-04)	0.003
Age (years)	1.01	(0.69; 1.48)	0.956
Cardiac index (l/min*1/m^2^)	0.12	(0.02; 0.42)	0.003
Reflection coefficient	1.56	(1.30; 2.00)	< 0.001

Data expressed as OR, 95% confidence interval (CI) and *p*-value. *HL* Hosmer–Lemeshow test, *OR* odds ratio, *AIx@75* Augmentation index corrected for heart rate of 75 bpm

### Males

An increase in TVR (*p* = 0.03), reflection coefficient (*p* < 0.01) and augmentation pressure (*p* = 0.01) in the univariate analysis of the group of male individuals represented a greater chance of having arterial stiffness (Table 3). Increasing age (*p* = 0.026) and cardiac index (*p* < 0.001) in the multivariate model were associated with a lower chance of having arterial stiffness, and an increase in reflection coefficient was associated with a higher chance (*p* < 0.001) (Table 4).

### Females

Among females, there was a lower chance of having arterial stiffness and an increase in cardiac output (*p* < 0.01), and a higher chance of an increase in the TVR (*p* < 0.01), in the reflection coefficient (*p* < 0.01), augmentation pressure (*p* < 0.01) and AIx@75 (*p* < 0.01) in the univariate analysis (Table 3). Furthermore, the increase in cardiac output was associated with a lower chance of having arterial stiffness (*p* = 0.003) in the multivariate model, while the increase in systolic volume and AIx@75 represented greater chances (*p*-values < 0.001 and 0.001, respectively) (Table 4).

### Children

An increase in reflection coefficient (*p* < 0.01) and augmentation pressure (*p* < 0.01) in the group of children in the univariate analysis were associated with a greater chance of having arterial stiffness (Table 3). An increase in reflection coefficient was associated with a higher chance of having arterial stiffness (*p* = 0.036) in the multivariate model, and an increase in cardiac output was associated with a lower chance (*p* < 0.001) (Table 4).

### Adolescents

The increase in cardiac output (*p* = 0.01) in the univariate analysis was less likely to present arterial stiffness, while the increase in TVR (*p* < 0.01), reflection coefficient (*p* < 0.01), AP (*p* < 0.01) and AIx@75 (*p* < 0.01) were more likely to have arterial stiffness (Table 3). In addition, the increase in cardiac index was associated with a lower chance of having arterial stiffness (*p* = 0.003) in the age-controlled multivariate model, while the increase in reflection coefficient represented a greater chance (*p* < 0.001) (Table 4).

## Discussion

In the present study, we evaluated the factors associated with reduced PPA in children and adolescents considered healthy, stratified by gender and age group. Participants with PPA < 1.49 were considered from the arterial stiffness group. An increase in the TVR, the reflection coefficient and the augmentation pressure in the univariate model were more likely to have arterial stiffness in all groups. The factors most likely to have arterial stiffness as assessed by the PPA in the multivariate model were the age increase and the reflection coefficient in the total sample, male group and in the child and adolescent groups. Moreover, age and AIx@75 were the factors most likely to present arterial stiffness in the female group. It is important to point out that the reflection coefficient measures the magnitude of the reflection wave, while the AIx@75 measures the percentage increase in the reflection wave in relation to the PPc.

PPA can be calculated in several ways: 1- difference between PPp and PPc (PPA = PPp—PPc), 2- relationship between PPp and PPc (PPA = PPp / PPc), 3- relationship between (PPp—PPc) / PPc and 4- absolute difference between SBPp and SBPc [6]. PPA in the present study was calculated using the PPp / PPc ratio. The minimum and maximum values were 0.95 and 2.05, respectively, and the median was 1.49. Population data from the Anglo-Cardiff Collaborative Trial (ACCT), obtained by a validated oscillometric device, suggest that PPA values, expressed as the brachial / aortic PP ratio, range from 1.7 in individuals younger than 20 years to 1.2 over 80 years old [28].

In comparing the coefficient of variation of the different PPA indices, we observed that the lowest coefficient of variation was the PPp / PPc ratio (12.54%). Furthermore, the PPp—PPc, (PPp—PPc) / PPc and SBPp – SBPc indexes were 38.87%, 36.89% and 46.67%, respectively.

PPA has been extensively studied in adults and the results show that PPA has a significant inverse relationship between age, and its reduction has a negative impact on mortality, cardiovascular events and on the damage to noble organs such as the heart, kidney and brain [1]. In contrast, PPA in children and adolescents is poorly studied, and the number of patients involved is very small. Mukarami et al. (2017a) were the first to study PPA in children and adolescents aged zero to 14 years (6.2 ± 3 years). These authors observed that contrary to what happens in adulthood, PPA increases during childhood. Mukarami et al. evaluated PPA in two ways: by the difference between PP in the descending aorta and PP in the ascending aorta, and by the PP descending / PP ascending ratio [11]. These same authors also showed a reduction in PPA in patients after aortic arch repair when compared to the control group [12] and after surgery for complete transposition of great arteries [13]. A limitation of these studies is that they were performed in anesthetized patients, making it difficult to compare them with studies performed in awake patients. A recent study by our group [14] showed that children and adolescents with type I diabetes had lower PPA when compared to children considered healthy, matched for gender and age. Similar to the present study, PPA in the type I diabetes group negatively correlated with total vascular resistance (TVR), augmentation pressure (AP), reflection coefficient and AIx@75 [14].

The variable age was included in the multiple regression analysis model in the present study, regardless of significance to control for effects. Age is the main non-modifiable factor associated with reduced PPA because normal vascular aging is the main causal factor for arterial stiffness of the great arteries [6, 28].

Increases in TVR, reflection coefficient, augmentation pressure and AIx@75 were more likely to have arterial stiffness in the univariate analysis of the entire studied sample. TVR is defined by the relationship between pressure and blood flow. The reflection coefficient is a measure of the magnitude of the reflection wave [28]. This index is determined by the relation between the amplitudes of the reflection and ejection waves, considered one of the main determinants of the AIx@75. Augmentation pressure is defined by the difference between the first systolic peak generated by the ejection wave (P1) and the second systolic peak (P2) which is attributed to the increase in SBPc caused by the reflected wave. In other words, the augmentation pressure corresponds to the part of the PPc that is added between the SBPc and the DBPc due to the increase in reflected wave [29]. On the other hand, the increase in cardiac output (Cardiac output = stroke volume x heart rate) and cardiac index (Cardiac output / body surface) presented a lower risk of arterial stiffness in the univariate analysis. In a multivariate model, only the increasing age and the cardiac index were associated with a lower chance of having arterial stiffness. On the other hand, an increase in the reflection coefficient was associated with a higher chance of increasing arterial stiffness, i. e. reduce the PPA.

Increases in TVR, reflection coefficient and augmentation pressure represented a greater chance of having arterial stiffness in the male group in the univariate analysis. Similarly to the total sample, increasing age and cardiac index were associated with a lower chance of having arterial stiffness in the multivariate model, and an increase in the reflection coefficient was associated with a higher chance of having arterial stiffness.

Univariate PPA analysis of the female and adolescent groups were similar. The increase in the cardiac output was associated with lower chance to have arterial stiffness, and increases in total vascular resistance (TVR), reflection coefficient and augmentation pressure, were associated with a greater chance of having arterial stiffness in adolescent group. In addition to these factors, the increase in AIx@75 in the female group also contributed to a greater chance of having arterial stiffness, that is, a reduction in PPA. In the multivariate model, an increase in cardiac output and age were associated with a lower chance of having arterial stiffness in the female group, while the increase in systolic volume and AIx@75 represented a greater chance of arterial stiffness. The AIx@75 is a measure, in percentage, of the increase in the augmentation pressure in relation to the PPc, which occurs due to the early return of the reflection wave [30]. An increase in cardiac index was associated with a lower chance of arterial stiffness in the multivariate model, and an increase in reflection coefficient was associated with a higher chance. In the multivariate model in the adolescent group, an increase in cardiac index was associated with a lower chance of having arterial stiffness, while the increase in age and reflection coefficient represented a greater chance of arterial stiffness.

Increases in reflection coefficient and augmentation pressure in the group of children were associated with a greater chance of having arterial stiffness. However, an increase in the cardiac index was associated with a lower chance of having arterial stiffness in the age-controlled multivariate model, while the increase in the reflection coefficient represented a greater chance of arterial stiffness.

The results of the present study show that the factors which present a higher risk of reduction in PPA in multivariate analysis logistic regression are related to the reflection wave. PWV, the gold standard for arterial stiffness, was not associated with a reduction in PPA. These results suggest that PPA is related to wave propagation velocity, reflected wave amplitude, reflection point, and the ventricular ejection duration and pattern. In contrast, aortic PWV represents intrinsic arterial stiffness. Furthermore, the influence of age is greater in AIx@75 than in aortic PWV before 50 years. Then, the influence of age after 50 years is greater on PWV [28]. PP significantly increases from the fifth decade onwards, suggesting that the rigidity of the great arteries predominantly occurs in the late phase of life [28]. Studies carried out in children and adolescents with Marfan syndrome reinforce these findings. Grillo et al. (2021) observed reduced PPA, although aortic stiffness, measured as PWV, seems to be the same as the general population. PPA was significantly and independently correlated with the aortic diameter in the Valsalva sinuses [31].

Studies show, in adults, that APP has a significant inverse relationship between age and its reduction has a negative impact on mortality, cardiovascular events and damage to noble organs such as the heart, kidney and brain [1]. Thus, longitudinal studies should be carried out with the aim of verifying the associations between PPA and cardiovascular outcomes, which, despite occurring in adulthood, subclinical changes begin early. As PPA reflects steady and pulsatile components of the regulation of the vessel response to blood pressure, this index has the potential to become an important marker for assessment of central hemodynamics and cardiovascular risk in different diseases [32].

### Study strengths and limitations

Our study has the following strengths. First; the sample was representative of the different regions of the Belo Horizonte city, gender, and age groups. The adjusted models considered the assumptions necessary to construct linear regression models (normal distribution, independence, and homoscedasticity of residues) and presented no multicollinearity problem. Second, we excluded volunteers with a significant respiratory history or medical diagnosis of asthma using the International Study of Asthma and Allergies in Childhood (ISAAC) questionnaire. Asthma is a prevalent disease in this age group. We [3] and other authors have already demonstrated an association of asthma and arterial stiffness in this population.

However, our findings present some limitations. First, due to its cross-sectional design, our study cannot be conclusive regarding the causal relationship between variables. Second, although the sample was adequate, it may have been too small for sub-group analysis. Third, we did not collect family history data for cardiovascular disease and dietary habits; and fourth, we did not exclude participants with a history of obstructive sleep-disorder breathing. We recently demonstrated that children with obstructive sleep-disorder breathing have increased arterial stiffness, evidenced by the increase in AIx@75 [33]. Herein, we show that the factors which present a greater chance of reducing PPA in the univariate logistic regression are related to the reflection wave, reflection coefficient, augmentation pressure and AIx@75.

## Conclusions

The results of the present study show that the factors that present a higher risk of reduction in PPA in univariate logistic regression are related to the reflection wave, meaning the reflection coefficient, augmentation pressure and AIx@75. PWV the gold standard for arterial stiffness was not associated with a reduction in PPA. PPA is related to wave propagation velocity, reflected wave amplitude, reflection point, and the ventricular ejection duration and pattern, while aortic PWV represents intrinsic arterial stiffness. Taken together, these data suggest that the assessment of these arterial stiffness indices, PPA and PWV, are not interchangeable, and that PPA, like AIx@75, may be a more sensitive marker in young individuals.

## Data Availability

The dataset analyzed during the current study is available from the corresponding author upon reasonable request.

## Abbreviations

AIx: Augmentation index
AIx@75: Augmentation index corrected by the heart rate of 75 bpm
BMI: Body mass index
CVD: Cardiovascular disease
HC: Hip circumference
HL: Hosmer–Lemeshow test
IPAQ-C: Physical Activity Questionnaire-Child
ISAAC: International Study of Asthma and Allergies in Childhood
OR: Odds ratio
Peds-QL 4.0: Pediatric Quality of Life Inventory Version 4.0
PP: Pulse pressure
PPA: Pulse pressure amplification
PPc: Central pulse pressure
PPp: Peripheral pulse pressure
PWV: Pulse wave velocity
SBPc: Central systolic blood pressure
SBPp: Peripheral systolic blood pressure
DBPc: Central diastolic blood pressure
DBPp: Peripheral diastolic blood pressure
MAP: Mean arterial pressure
RC: reflection coefficient
AP: Augmentation pressure
TVR: Total vascular resistance
WC: Waist circumference
