# Correction: Factors associated with arterial stiffness assessed by pulse pressure amplification in healthy children and adolescents: a cross-sectional study

**DOI:** 10.1186/s12887-023-04052-8

**Published:** 2023-05-09

**Authors:** Leticia Pereira Salomão, Giselle Santos Magalhães, José Felippe Pinho da Silva, Luzia Maria dos Santos, Isabel Cristina Gomes Moura, Bruno Almeida Rezende, Maria Glória Rodrigues‑Machado

**Affiliations:** Programa de Pós-Graduação em Ciências da Saúde – Faculdade Ciências Médicas de Minas Gerais, Alameda: Ezequiel Dias, N 275. Bairro: Centro, CEP 30130‑110 Belo Horizonte/MG, Brasil

**Correction:** **BMC Pediatr 23, 154 (2023)**

**https://doi.org/10.1186/s12887-023-03942-1**

Following publication of the original article [1], the author reported the the arrow in Fig. 1D is slightly misplaced. It should be as presented below.

**Fig. 1:**
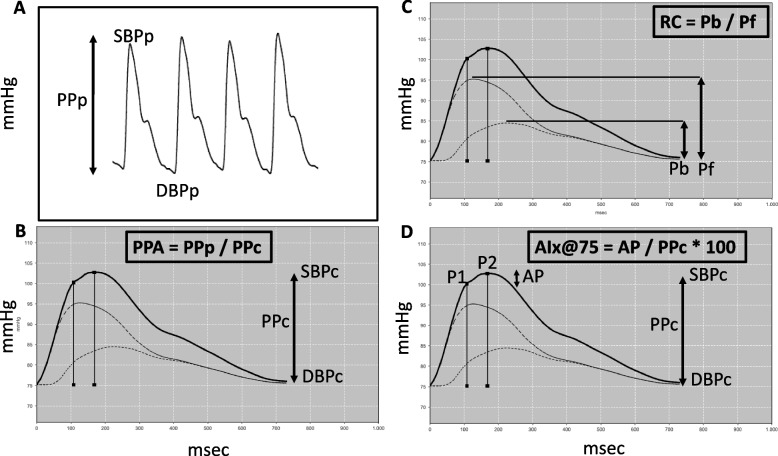
Pulse waves from the brachial (**A**) and aortic (**B**‐**D**) arteries in a female teenager. **A** Peripheral pulse wave (measured). SBPp and DBPp = Peripheral systolic and diastolic blood pressure; PPp = Peripheral pulse pressure. **B** Central pulse wave (calculated). SBPc and DBPc = Central systolic and diastolic blood pressure; PPc = Central pulse pressure; PPA = Pulse pressure amplifcation (PPp / PPc). **C** Pf = Forward or ejection wave; Pb = Backward or refection wave; RC – Refection coefficient. **D** P1 = First systolic peak; P2 = Second systolic peak, AP = Augmentation pressure (P2 – P1), AIx@75 = Augmentation index corrected for a heart rate of 75 bpm

The original article has been updated.
